# Bacterial Stigmergy: An Organising Principle of Multicellular Collective Behaviours of Bacteria

**DOI:** 10.1155/2015/387342

**Published:** 2015-01-08

**Authors:** Erin S. Gloag, Lynne Turnbull, Cynthia B. Whitchurch

**Affiliations:** The ithree Institute, University of Technology Sydney, P.O. Box 123, Broadway, Sydney, NSW 2007, Australia

## Abstract

The self-organisation of collective behaviours often manifests as dramatic patterns of emergent large-scale order. This is true for relatively “simple” entities such as microbial communities and robot “swarms,” through to more complex self-organised systems such as those displayed by social insects, migrating herds, and many human activities. The principle of stigmergy describes those self-organised phenomena that emerge as a consequence of indirect communication between individuals of the group through the generation of persistent cues in the environment. Interestingly, despite numerous examples of multicellular behaviours of bacteria, the principle of stigmergy has yet to become an accepted theoretical framework that describes how bacterial collectives self-organise. Here we review some examples of multicellular bacterial behaviours in the context of stigmergy with the aim of bringing this powerful and elegant self-organisation principle to the attention of the microbial research community.

## 1. Introduction

The emergence of self-organised patterns in living and nonliving systems has fascinated scientists for centuries. In biological systems, the coordination of group behaviours and the subsequent emergence of large-scale patterns are inherently more complex than that which spontaneously emerges in nonliving systems [1], involving an interplay of physical, chemical, and biological interactions, both physiological and behavioural, that have been honed through natural selection [2–4]. Many self-organised phenomena in both biotic and abiotic systems can be explained by the principle of stigmergy, a concept that describes self-organised systems that arise through an individual of the collective influencing the movement or behaviour of other individuals at a later point in time through the generation of persistent cues within the environment [5, 6].

The concept of stigmergy was first introduced by the entomologist Grassé in 1959 to explain the construction of termite colonies [5]. This powerful concept, for the first time, explained how apparently random and independent movements of an individual could result in the transfer of persistent information locally, thereby manifesting as coordinated behaviour at a global level [2, 6]. The principle of stigmergy has since been employed to describe a vast array of group activities such as the laying-down of pheromone trails by foraging ants, herd migration in animals, and various aspects of human activities including the following of hiking trails and pedestrian footpaths [7–11] as well as artificial systems such as “swarm intelligence” within robotics and computing [12–17]. Interestingly, even the development of multicellular tissues has been described as a stigmergic phenomenon in which chemical cues are embedded in extracellular matrix material [18]. As other scientific fields such as social sciences, technology, and computer sciences began adopting the concept of stigmergy to help describe and explain various phenomena of emergent behaviour or properties, various types or categories of stigmergy have been described in an attempt to better understand the different stimuli and responses which influence the stigmergic interactions of the agents in these systems.

Sematectonic and marker-based stigmergy differentiate between the forms of communication, that is, the types of signals that initiate a response or behaviour change [15, 16, 19]. Sematectonic stigmergy was first coined by Wilson and describes communication through physical changes to the environment [19], for example, the following of trails by herd animals, pedestrians, and hikers [7] and the construction of wasp nests, where the development of the physical structure acts as cues for the next steps or process in the design [6, 20]. In contrast, marker-based stigmergy refers to communication through the deposition of chemical signals within the environment [15, 16, 19], for example, the following of pheromone trails by ants aiding in their food foraging behaviour [21]. A further distinction between these two variations is that for sematectonic stigmergy the communicative information tends to provide a direct contribution to the task/emergent property, whereas in marker-based stigmergy the cues do not take direct action but rather influence subsequent behaviour, stimulating self-organisation for its effective completion [15].

Quantitative and qualitative stigmergy are other forms that were introduced by Theraulaz and Bonabeau to describe the different stimulus signalling and response outcomes [6]. Quantitative stigmergy describes a system where an individual's response to a stimulus intensifies the stimulus, with the nature of these stigmergic systems often leading to positive feedback [2, 6]. Here again the following of pheromone trails by ants provides an example of quantitative stigmergy, whereby continuous ant traffic along specific pheromone trails results in the deposition of more pheromone, thereby amplifying the signal and attracting further ants to these trails, which in turn lay down more pheromone. Qualitative stigmergy refers to self-organising systems that arise from an individual responding to a stimulus that in turn creates a qualitatively different stimulus, thereby triggering a separate response [2, 6]. The building of a wasp nest provides an example of qualitative stigmergy as the growing structure provides different signals and cues based on the stage of construction and results in distinct building behaviours [2, 6, 20]. It has also been recognised that both sematectonic and marker-based signals can initiate either quantitative or qualitative responses [16]. Finally, passive and active stigmergy have been described; however, as these two variations have for the most part been applied only to collective swarm intelligence in robotics [12, 22], they will not be discussed here.

Whilst there are many examples of self-organised multicellular behaviours of bacteria, the concept of stigmergy has rarely been used to describe these phenomena. Here we review some examples of bacterial collective behaviours that may be described in the context of the organising principle of stigmergy.

## 2. Bacterial Swarms

Many species of bacteria are able to actively migrate across surfaces via a number of different mechanisms including twitching, gliding, and flagella-mediated swarming motilities. These motilities can facilitate the surface translocation of individual cells but can also manifest as highly organised multicellular “swarms” that enable rapid expansion of the bacterial communities. Here we show that stigmergy explains many of these “swarming” behaviours of bacteria.

**Figure 1:**
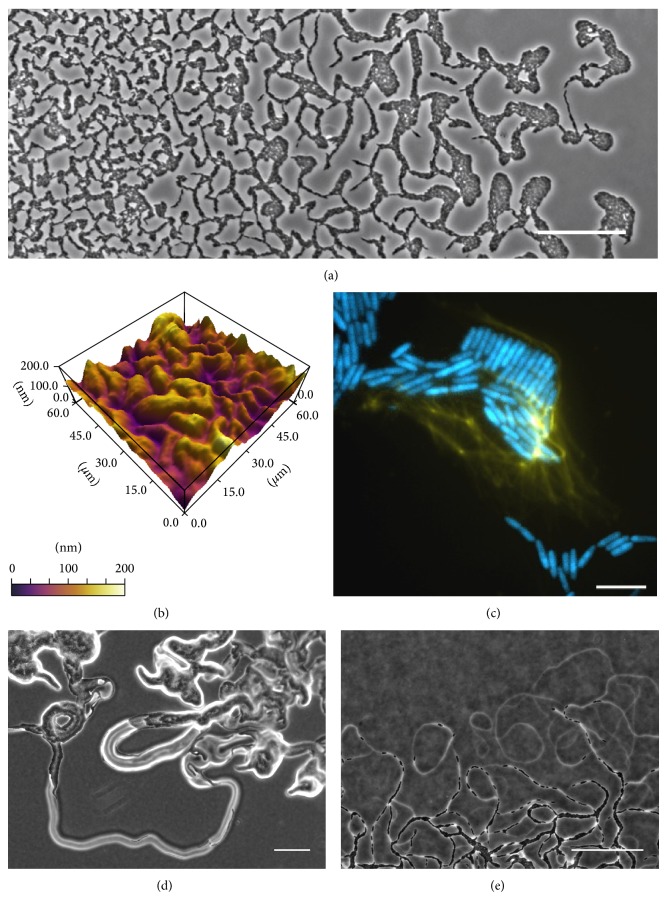
Stigmergic self-organisation of bacterial communities. (a)* Pseudomonas aeruginosa* interstitial biofilm imaged using phase contrast microscopy depicting the emergent pattern formation. At the advancing edge are rafts of cells that initiate biofilm expansion, behind which there is an interconnected lattice-like network of cellular trails. Scale bar indicates 50 *μ*m. (b) 3D rendered image of the interconnected furrow network underlying the* P. aeruginosa* interstitial biofilms imaged using atomic force microscopy (AFM) within the lattice-like network. Height scale is relative. (c)* P. aeruginosa* expressing cyan fluorescent protein (CFP; blue) interstitial biofilms were grown on media supplemented with the cell impermeant nucleic acid dye TOTO-1 to visualize eDNA (yellow) and imaged using OMX BLAZE wide-field microscopy. Scale bar indicates 5 *μ*m. Swarming communities of (d)* Pr. vulgaris* and (e)* M. xanthus* grown on semisolid nutrient media and imaged using phase contrast microscopy revealing the phase bright trails routinely observed at the leading edge. Scale bar is 100 *μ*m.

Twitching motility is a mechanism of surface translocation that is powered by the extension, surface binding, and subsequent retraction of type IV pili (tfp) [23, 24]. Under appropriate conditions, twitching motility is as a complex, highly coordinated multicellular behaviour that leads to the active expansion of the bacterial community across solidified nutrient media [25–27]. It has been found that when* Pseudomonas aeruginosa* is cultured at the interface of nutrient media that has been solidified with 0.8% gellan gum and an abiotic surface such as plastic or glass, twitching motility can lead to the formation of highly structured multicellular communities at the interstitial space. These have several characteristic micromorphological features including large vanguard rafts of highly aligned cells at the leading edge behind which there is an intricate, interconnected lattice-like network of trails of cells (Figure 1(a); [25]). Semmler et al. proposed that as the vanguard rafts migrated across the surface of the semisolid nutrient media, they created some form of trail along which ensuing cells preferentially followed [25].

We have shown recently that the emergence of the interconnected network of trails is likely to occur due to the formation of an interconnected furrow system in the underlying semisolid media (Figure 1(b); [28, 29]). We recently applied the concept of stigmergy to describe the emergent self-organisation of* P. aeruginosa* interstitial communities that occurs as a consequence of cells creating and travelling within the furrow network [29]. To our knowledge this was the first description of stigmergic behaviour driven by physical cues within the environment. We hypothesised that as cellular aggregates migrated across the media surface, they forged a furrow along which ensuing cells migrated and in doing so continued to remodel the substratum thereby refining the furrow system [28, 29]. We proposed that the furrows physically confine cells thereby directing cell movement and contributing to the emergence of the intricate interconnected network of cellular trails that are a characteristic feature of these biofilms (Figure 1(a); [28, 29]). This is an example of sematectonic and quantitative stigmergy and is highly reminiscent of the physical trail following displayed by animals during herd migrations and by humans following hiking trails and pedestrian footpaths [7–9, 15].

Interestingly, some bacteria from diverse genera display an “agar pitting” phenotype which can be used as an identifying feature for these species [30–35]. One such organism,* Dichelobacter nodosus*, also produces striking interconnected pattern networks reminiscent of that of* P. aeruginosa* when they are grown at the interstitial space between the petri dish and media [36]. However, whether this emergent pattern arises due to the corrosion of the agar during biofilm expansion, creating furrows that guide the movements of the bacteria remains to be determined. The agar pitting phenotypes of both* D. nodosus* and* Moraxella bovis* have been correlated with the presence of tfp. It has been speculated that the agar polysaccharides may act as ligands to which the tfp bind and that the physical interaction of the tfp with the agar may be responsible for the agar pitting phenotype [32]. It is interesting to speculate that the formation of furrow networks may constitute a more global mechanism for the stigmergic organisation of bacterial communities.

We have recently also identified a role for extracellular DNA (eDNA) in coordinating the collective behaviour of* P. aeruginosa* cells undergoing twitching motility-mediated biofilm expansion [28]. We observed that* P. aeruginosa* interstitial biofilms contain eDNA distributed either as a fine coating of the cells or as concentrated, punctate foci from which thin tendrils radiated in the overall direction of the motion of cells (Figure 1(c); [28]). Removal of this eDNA, through the incorporation of the eDNA-degrading enzyme DNaseI into the solidified nutrient media, resulted in the abrogation of the characteristic interconnected pattern network of these biofilms [28].

To understand the role of eDNA within these biofilms we employed a computer vision and cell tracking analysis pipeline [28, 37, 38] to quantitate the behaviour of the individual cells in the absence and presence of DNaseI. Interrogation of the resulting image informatics database revealed that eDNA facilitates twitching motility-mediated biofilm expansion by enabling more frequent movements of individual cells, thereby resulting in more sustained motion and greater distances traversed by individual cells over longer time periods. These analyses also revealed that eDNA is required for maintaining coherent cell behaviour and cell alignment over time [28]. Previous reports have identified that* P. aeruginosa* tfp bind to DNA [39] and that* P. aeruginosa* cells spontaneously pneumatically orient with the direction of extended DNA chains in a matrix of aligned, concentrated DNA [40]. We proposed that the bed of aligned eDNA molecules within* P. aeruginosa* interstitial biofilms maintains cell orientations by aligning cells to the thin strands of eDNA and that eDNA provides a substrate for optimal tfp binding, consequently enabling more frequent tfp-powered translocations, ensuring smooth traffic flow within the trail network and a consistent supply of cells to the leading edge of the expanding biofilm [28]. We also propose that eDNA serves as an intercellular “glue” that binds the cells together within vanguard raft assemblages thereby facilitating coherent cell movements to power migration of leading edge rafts into virgin territory [28].

The ability of eDNA to promote cohesive group behaviour during active biofilm expansion is an example of sematectonic stimergy. It could be further argued that the redistribution of eDNA through the biofilm is also an example of quantitative stigmergy as continued cellular migration through the concentrated regions of eDNA results in the production of fine tendrils of eDNA aligned with the direction of bacterial migration which then directs and maintains the alignment of ensuing cells along these eDNA strands thereby maintaining traffic flow in the overall direction of travel of the preceding cells [28].

Flagella-mediated swarming motility of* Proteus* spp. leads to the formation of rapidly expanding colonies grown on agar that are characterised by a repeated concentric circle pattern that extends across the swarm. This patterning is attributed to continuous rounds of cell differentiation, where the normal rod cells, which are largely nonmotile, differentiate into long, hyperflagellated swarmer cells. As a collective these swarmer cells rapidly migrate across the surface until they differentiate back to the nonmotile normal cells resulting in consolidation and the formation of the observed ring pattern [41, 42]. The flagella of* Proteus* swarmer cells interweave with flagella from the same cell and with those of neighbouring cells, forming a connected and highly synchronised swarming front that aids in the rapid expansion by these colonies [43]. The secretion of an extracellular slime has been found to facilitate the collective swarming behaviour of* Pr. mirabilis*. At the leading edge of* Pr. mirabilis* swarms, swarmer cells are encased in a slime layer and appear to preferentially move along an interconnected network of phase bright slime trails (Figure 1(d); [44]). It has been hypothesised that the slime trails aid in directing swarming motility and the slime encasement facilitates the maintenance of a cohesive organisation of cells [42, 44]. Therefore slime production and slime trail following promote the self-organisation of collective behaviours necessary for the expansion of the swarming colony.

Gliding motility of* Myxococcus xanthus* is mediated by the combined efforts of two motility modes; social (S) motility and adventurous (A) motility. Similar to twitching motility, S-motility is driven by the extension, binding, and retraction of tfp with this motility mode being typically displayed by groups or clusters of cells [45, 46]. A-motility mediates single cell migration and in contrast to that of S-motility, the machinery driving A-motility is yet to be confirmed and is an area of controversy [47, 48]. However all current schools of thought predict the role of a secreted slime in facilitating the A-motility of this organism [49–51], where phase bright trails are observed at the leading edge of the* M. xanthus* swarms when grown on semisolid media (Figure 1(e); [52]), similar to that of* Pr. mirabilis*.* M. xanthus* cells preferentially migrate along these slime trails, with cells frequently observed to turn onto the trails rather than migrating across virgin territory. Continued cellular traffic along the trails results in their thickening and extension as a consequence of continued slime deposition [51, 53]. It is recognised that this trail following behaviour coordinates the collective behaviour of* M. xanthus* cells, specifically those displaying A-motility, at the leading edge of the surface swarms, and contributes to the emergence of the interconnected pattern networks at these areas [51–53].

The following of slime trails during* Pr. mirabilis* swarming and* M. xanthus* gliding motilities are both sematectonic and quantitative stigmergic systems, where the stimulus (slime) is a physical manifestation within the environment that directly contributes to the expansion of the community as it is required for the motility of the organism. This is particularly the case for the slime mediating the A-motility of* M. xanthus* (sematectonic stigmergy). Continued traffic along the slime trails amplifies the slime deposited resulting in further recruitment of cells migrating along these regions (quantitative stigmergy).

It has been shown recently that the formation of vortexes comprised of thousands of bacteria rotating in unison that occur during active surface migration by* Paenibacillus vortex* biofilms occurs as a consequence of the actions of a subpopulation of filamentous cells that direct the motion of the other members of the collective [54, 55]. This appears to be another example of bacterial stigmergy, though it remains to be determined if this collective behaviour occurs as a consequence of physical alteration of the environment, slime, or chemical cues.

A number of computational models have been developed to describe collective behaviours displayed during swarm activities, particularly for* M. xanthus* [56–58]. Due to the inherent difficulties in modelling biological systems, a number of these models do not truly reflect experimental observations or contain artefacts as a consequence of the rule parameters incorporated into the model [59]. Stigmergic systems have long been the focus of extensive computational modelling to understand the emergent properties within these systems [7–10] and to relate stigmergic principles from one system to another in an attempt to draw comparisons from well-studied and established systems [17]. It is our contention that a similar approach could be taken for modelling bacterial swarming communities through the incorporation of key ideas from other stigmergic models, such as those of Helbing et al. and Goldstone et al., who modelled sematectonic and quantitative stigmergic systems such as trail following by humans and animals [7–9]. Incorporating such an integrated approach could potentially yield further insight into the self-organisation and emergent pattern networks of bacterial swarms.

## 3. Bacterial Biofilms

Bacterial biofilms are multicellular communities of bacteria that are attached to each other and often a biotic or abiotic surface via a self-produced extracellular matrix comprised of extracellular polymeric substances (EPS) including exopolysaccharides, eDNA, proteins, and lipids [60–62]. The production of this EPS matrix is essential for biofilm development as it provides intercellular connectivity that binds cells to each other and, in the case of surface-attached biofilms, provides surface adherence [60, 61]. The ability of the EPS matrix produced by biofilm cells to promote cohesion and surface attachment of the biofilm community is an example of sematectonic stigmergy.

It has been observed that individual* P. aeruginosa* cells undergo extensive twitching motility-mediated surface exploration prior to subsequent microcolony formation during the early stages of biofilm formation on glass submerged in liquid nutrient media [63–66]. Zhao and colleagues showed recently that, during surface exploration,* P. aeruginosa* cells deposited trails of the exopolysaccharide Psl, which appeared to recruit additional cells along these trails leading to a positive feedback loop of further Psl deposition and subsequent cell attraction [67]. It was hypothesised that this trail following behaviour was facilitated by twitching-motility-mediated surface exploration, where the tfp were thought to probe the surrounding areas for Psl networks, promoting binding of the tfp and directing cellular migration to these areas [67]. In areas of high Psl concentration, cells were observed to adhere to the substratum and correlated to the subsequent sites of microcolony formation [67, 68]. This mechanism of following exopolysaccharide trails to coordinate the single cellular motilities of* P. aeruginosa* during early biofilm development is an example of sematectonic and quantitative stigmergy. Zhao et al. used a “rich-getting-richer” analogy comparable to that of capitalist economies to describe this emergent behaviour [67], which has itself been described as a stigmergic system [15, 16].

## 4. Quorum Sensing

In many bacterial communities quorum sensing regulates and coordinates social behaviours, such as bioluminescence, secretion of public goods, and the switch from planktonic to the biofilm mode of growth [69]. Quorum sensing occurs through the release of small molecules by individual bacteria into the environment by passive diffusion. The concentration of these small molecules increases within the environment with increasing cell density, permitting cells to gather information about their surrounding neighbours. Once a sufficient quantity of signal is present within the environment, reflecting a critical population density, a gene regulation cascade is initiated culminating in the up- or downregulation of the expression of various genes required for social behaviours, virulence factor production, and so forth [69, 70]. In this manner it has been identified that quorum sensing can regulate the expression of over 300 genes within* P. aeruginosa* [69].

Quorum sensing within bacterial communities bares a striking resemblance to pheromone signalling that coordinates the collective behaviours of social insects. It is therefore interesting to speculate whether quorum sensing offers another example of stigmergic self-organisation within bacterial communities. Under circumstances where quorum sensing signalling molecules are able to persist and accumulate within the environment, then quorum sensing could be considered an example of marker-based stigmergy whereby the release of signalling molecules into the environment stimulates collective behaviours of the growing bacterial population, similar to the pheromones coordinating the social behaviours of ants and termites. It could be suggested that quorum sensing, in addition to marker-based stigmergy, is also an example of qualitative stigmergy, where, depending on their concentration, the quorum sensing signals trigger different responses by the bacterial population.

## 5. Summary and Future Directions

We have presented a number of examples in which bacteria employ stigmergic self-organisation to coordinate their collective behaviours and found that sematectonic and quantitative stigmergic systems in the form of trail following were the most prevalent in the above examples. This highlights the conserved nature of self-organising mechanisms within nature regardless of the cognitive abilities of the individual entities and suggests a common evolution of trail following as a simple yet effective means of coordinating collective behaviours.

The idea that self-organising systems utilised by bacterial communities are similar to those utilised by higher organisms is gaining interest within the scientific community. A recent review has called for the employment of a more integrative approach across scientific fields in the study of self-organising systems [71]. Stigmergy provides an excellent example of this approach where, since its first introduction within the field of entomology [5], the importance of this concept has been recognised across diverse areas ranging from biology to social sciences, technology, and computer sciences [15, 16, 72]. The wide acceptance of stigmergy can, for the most part, be attributed to a special edition of* Artificial Life* dedicated to stigmergic systems [6, 73], with the hopes of bringing this concept to the forefront within the scientific community. This concept, despite its obvious application to the understanding of multicellular bacterial behaviours, has been largely overlooked within the field of microbiology.

Here we recognise the importance of the concept of stigmergic self-organisation and the implications it has on understanding the collective behaviours of complex multicellular bacterial communities. We propose that bacterial stigmergy should be included in the repertoire of systems that bacteria employ to control multicellular activities. Furthermore, we suggest that bacterial stigmergic systems may provide testable models to explore stigmergic self-organisation at a molecular level [29], which is currently an unexplored concept and will ultimately lead to greater understanding of other biological stigmergic systems. Understanding the mechanisms employed by bacteria to coordinate their multicellular behaviours may lead to the development of novel strategies to control infections and biofouling in industrial and marine settings.
